# Stress, eating, and the structure of time in ADHD: A phenomenological perspective on ecological momentary assessment

**DOI:** 10.1016/j.nsa.2026.106987

**Published:** 2026-02-10

**Authors:** Stefan Jerotic

**Affiliations:** Clinic for Psychiatry, University Clinical Centre of Serbia, Belgrade, Serbia; Faculty of Medicine, University of Belgrade, Belgrade, Serbia

Ruf et al. (2025) have pioneered the use of ecological momentary assessment (EMA) to investigate stress-induced eating in adults with attention-deficit/hyperactivity disorder (ADHD). In their 3-day EMA study, adults with ADHD reported stress and impulsivity multiple times per day, also logging food intake; interestingly, no link was found between momentary stress and the occurrence or amount of eating. Neither trait nor state impulsivity significantly moderated the stress–eating relationship. These null findings, while preliminary (the authors note the small sample and brief sampling period as limitations), raise important questions about how experience is being captured, both in ADHD and more broadly.

From a phenomenological standpoint, ADHD is not merely a checklist of symptoms but a distinct way of being-in-the-world; a fundamental alteration in an individual's experience of time, body, and social relation. Temporality seems especially central. Classic models have proposed that ADHD minds are in a sense “blind” to the past and future, operating under the tyranny of the present moment (Barkley, 1997). Furthermore, empirical research supports the argument that changes in the perception of time are in fact a focal feature of adult ADHD (Weissenberger et al., 2021), even suggesting that temporal distortions may be at the root of ADHD related deficits. This resonates with insights from phenomenological psychiatry: patients often describe being out of sync with the world's clock, perpetually late or hurried, as if their inner tempo misaligns with expected rhythms. One study even introduced the concept of “own-time spaces” – situations where individuals with ADHD can harmonize with their personal rhythm and in which ADHD symptoms recede (Rasmussen et al., 2025). This indicates that temporality is not an incidental backdrop but a core facet of the lived experience in ADHD.

One of the most important tensions in the methodology of EMA and phenomenology is the question of time. EMA samples “moments” of experience – in Ruf et al.’s study, eight signals a day over three days. Implicit in this approach is an assumption that psychological phenomena may be understood as a series of discrete states across time. If a stress impulse and an eating episode were separated by 20 min, whether EMA records them as “linked” depends on timing and the person's perception. So, non-findings should be interpreted with care given these methodological constraints. None of this is to diminish EMA's value – only to temper our confidence in what EMA cannot tell us. Human experience has a narrative texture that momentary snapshots alone cannot reproduce. As phenomenologist Karl Jaspers argued, we need both *Erklären* (explanation by objective factors) and *Verstehen* (understanding the subjective meaning) to fully grasp psychiatric phenomena (Gough, 2023; Jerotic, 2021). EMA is a powerful tool for *Erklären* – it helps establish temporal sequences and correlations in daily life. Phenomenological analysis such as the analysis of temporality contributes to *Verstehen* – helping us to ask, among other things, what is the meaning of these observations for the person living through them?

Phenomenologically adequate descriptions of individual experience are notoriously difficult to obtain, even though recently instruments measuring temporality pre-loaded with phenomenological concepts have been developed and used in clinical practice (Jerotic et al., 2025; Stanghellini et al., 2022; Szuła et al., 2024). Even so, describing temporality extensively, often requires in-depth phenomenological interviews, and patients often struggle to articulate these experiences. Thus, phenomenological analyses of time-consciousness occupy a position at the very opposite pole from conventional static measures, which remain tied to broadly accepted “symptoms.” Although phenomenological concepts are not explicitly integrated into their methodology, EMAs may be seen as occupying an intermediate position in this hierarchy, helping to bridge the gap between static measures and phenomenological analysis.

Principles coming from phenomenology and enactive psychiatry remind us that people with ADHD are active agents continually adapting to their environments. They develop creative workarounds, seek niches or “own-time spaces”, and often possess unique strengths (e.g. hyperfocus on tasks of great interest, high energy, spontaneity) that a purely deficit-focused metric would overlook (Ginapp et al., 2022). Ruf et al.’s study, for example, hints that adults with ADHD are not uniformly vulnerable in the way we assumed; they may be handling certain challenges (like stress eating) better than expected.

In conclusion, Ruf et al. (2025) have provided a valuable, ecologically valid window into daily-life processes of ADHD, and their use of EMA exemplifies the kind of methodological innovation needed to bridge laboratory findings with the lifeworld of patients. The data, if interpreted with phenomenological sensitivity, reinforces that ADHD is a condition of complex dissonances, which can be interpreted through the lens of temporality. EMA's strength is capturing the flicker of these dissonances in real time, revealing patterns (or absences of patterns) that static assessments miss. The ideal approach going forward may be a kind of a synthesis: using EMA to gather ecologically valid evidence, but embedding it in a framework of understanding that considers phenomenological aspects such as embodiment and temporality.

## Conflicts of interest

None.
